# Tackling CASMI 2012: Solutions from MetFrag and MetFusion

**DOI:** 10.3390/metabo3030623

**Published:** 2013-08-05

**Authors:** Christoph Ruttkies, Michael Gerlich, Steffen Neumann

**Affiliations:** Leibniz Institute of Plant Biochemistry, Department of Stress and Developmental Biology, Weinberg 3, DE-06120 Halle (Saale), Germany; E-Mail: sneumann@ipb-halle.de

**Keywords:** mass spectrometry, metabolite identification, MetFrag, MetFusion, metabolite likeness, molecular formula

## Abstract

The task in the critical assessment of small molecule identification (CASMI) contest category 2 was to determine the identification of (initially) unknown compounds for which high-resolution tandem mass spectra were published. We focused on computer-assisted methods that tried to correctly identify the compound automatically and entered the contest with MetFrag and MetFusion to score candidate structures retrieved from the PubChem structure database. MetFrag was combined with the metabolite-likeness score, which helped to improve the performance for the natural product challenges. We present the results, discuss the performance, and give details of how to interpret the MetFrag and MetFusion output.

## 1. Introduction

The critical assessment of small molecule identification contest (CASMI) was organised in 2012 by Emma Schymanski and Steffen Neumann, to call upon the computational mass spectrometry community and demonstrate the performance of compound identification from mass spectrometry data. 

At the Leibniz Institute of Plant Biochemistry (IPB), we are developing several tools for metabolite identification. The MetFrag system [1] is able to perform *in silico* fragmentation of candidate structures, which can be retrieved from compound databases or obtained through structure generation [2]. The IPB is also part of the MassBank consortium [4], which collects a large number of reference spectra, particularly of soft electrospray ionisation (ESI) spectra. Our MetFusion system [5] integrates these two strategies to obtain a more reliable identification compared to each individual approach taken alone. 

In the CASMI contest, our tools did not officially take part because one author was in the organisation team and some of the challenge spectra were obtained at the IPB. Nevertheless, we tried to approach the challenges in as unbiased a manner as possible, and did not use our inside knowledge to tune any parameters in order to obtain better results. We also restricted the participation to category 2 (“best structure identification for high resolution liquid chromatography/mass spectrometry (LC/MS) data”) and did not submit the molecular formulas to category 1 (“best molecular formula for high resolution LC/MS data”). 

## 2. Methods

The spectra preprocessing steps and the elimination of redundant candidate structures are the same for both MetFrag and MetFusion. 

### 2.1. Spectra Processing and Neutral Mass Heuristics

All of the challenges were measured in a single ionization mode, but with multiple ionization energies. If a challenge provided two or more spectra, the spectra were merged to create a corresponding composite spectrum. This processing step was recommended by the MassBank consortium [4] for a more reliable identification. Challenges 2, 10 and 12 each consisted of only one spectrum, so the spectra merging was not applied to them. We used the mzClust grouping algorithm in xcms (version 1.37.0) [6,7]. The composite spectrum contains the unique peaks where m/z values are averaged and the maximum intensity across all spectra is used. The R-code for the merging is shown in Appendix B. 

To determine the neutral mass of a compound, we used a simple heuristic which located the lowest m/z in the isotope pattern as a monoisotopic peak and then removed the adduct, taking the polarity of the measurement into account to automatically deduce the neutral exact mass of the compounds for the candidate search. 

### 2.2. Eliminating Redundant Candidates

Both MetFrag and MetFusion obtain candidate structures from chemical databases. They often contain redundant structures which increase the candidate lists without adding chemical diversity. In addition, mass spectrometry can, in general, not distinguish between the stereoisomers of a compound and the identification methods we use assign identical scores to isomers. Therefore, we eliminate redundant candidate structures with an InChIKey-based filtering. 

The InChIKey is a string that is characteristic of the molecular structure, where the first block of 14 characters is determined by the molecular skeleton (or connectivity). More information regarding both InChI and InChIKey can be found elsewhere [8]. We calculate the InChIKey for each candidate and keep only candidates with a unique first InChIKey block. 

### 2.3. In silico Fragmentation with MetFrag

We used MetFrag as described in Wolf *et al.* [1], with the composite spectra as explained in Section 2.1 to submit candidates for all challenges in CASMI category 2. We queried a local PubChem [3] mirror (created September 2010) for the candidate retrieval and filtered as explained in Section 2.2. For the candidate selection we used the putative neutral exact mass and a mass window of 5 ppm and 0.001 Da mass deviation for the fragment matching. For later resubmissions for Challenge 5, we adapted the mass window to 10 ppm and 0.002 Da for, due to the higher mass error. For this paper, we additionally used a molecular formula candidate search using the correct formulas which were not known during the contest but given in the solutions. This allows estimation of the MetFrag performance the correct molecular formulas are used as input. 

The score calculated by MetFrag evaluates the match of *in silico*-generated fragments of the candidate molecules to the given challenge tandem mass spectra The mass as well as the intensity of the peak matched by a fragment are considered in the score. 

Compounds for challenges 1 to 6 were known to be natural products, as explained on the CASMI website. Because large compound databases, such as PubChem [9], contain many non-natural compounds, several filtering strategies have been developed for metabolomics data. While Kind and Fiehn [10] proposed filter criteria based on the molecular formula, Peironcely *et al.* [11] used machine learning to train a random forest model [12] on metabolite structures from the Human Metabolome Database (HMDB) [13] and structures from the ZINC database [14] to predict a metabolite-likeness score (MLS) based on structural fingerprints. 

We used the MLS to prefer biological compounds for challenges 1–6. For those challenges, we used the adapted version of the final score:

*Score_final_* = *Score_MetFrag_* + *ω* · *MLS*

to obtain the ranking, where *ω* represents the weight of the MLS which we arbitrarily set to 0.5 to give it a lower influence in the final score than the MetFrag score. In the future we plan to optimise *ω* by learning from given data. The influence of the metabolite-likeness score on the rankings of candidates was investigated by comparing the rankings of results with *ω* =0 and *ω* =0.5. 

### 2.4. MetFusion: Integration of MetFrag with Spectral Libraries

We also applied MetFusion [5] to generate submissions for all Category 2 challenges. We used the MassBank spectral library and PubChem compound database, which in this case was queried online in January and March 2013. For the candidate selection we used the putative neutral mass and a mass window of 10 ppm. A mass window of 10 ppm is sufficient as all Category 2 challenges promise an accuracy of <10 ppm. For the fragment matching, we applied a window of 0.002 Da and 10 ppm. As explained above, we used composite query spectra and the InChIKey-based candidate filtering. 

MassBank provides separate search forms for either a precursor mass search or peak list search. The combination of both types of information is currently not available, although it would be possible to search MassBank with both an MS/MS spectrum and explicitly apply the precursor neutral mass as filter afterwards. This search strategy is used by, e.g., the Metlin database. Instead, MetFusion invokes the peak list search, so MassBank will also return compounds with similar MS/MS spectra in order to possibly return also structurally similar compounds. MetFusion then implicitly combines the fragmentation similarity from MassBank with the exact mass hit from PubChem. 

All challenges were queried against all available ESI spectra in MassBank [4]. For the resubmissions, we also included instruments with ion sources at atmospheric-pressure levels, namely chemical ion­ization (APCI) and photoionization (APPI). This instrument selection covers triple quadrupole (QqQ), quadrupole time-of-flight (QTOF) and Orbitrap devices i.e., both nominal and accurate mass spectra were queried. 

Besides the peak list and instrument selection, the number of result hits and the intensity cut-off are the only parameters for the MassBank peak search. The result limit was set to 100 hits and the intensity cut-off was set to 5. The intensity cut-off determines which peaks are ignored due to having a lower intensity than the specified cut-off. MassBank internally applies a fixed 0.3 Da mass window when matching peaks. MassBank also utilizes the intensity information for spectra comparison, i.e., low intensity peaks have less weight in the resulting scores. 

For the MassBank query results, we also performed an InChIKey-based filtering where among the duplicates only the entry with the best MassBank score, i.e., the highest spectral similarity, was kept. The MetFusion workflow and the scoring have been described earlier [5]. 

In the next section we also discuss the chemical similarity, e.g., between the correct solution and the most similar MassBank record. We used the Tanimoto similarity based on the fingerprints of the structures as implemented in the CDK [15]. A Tanimoto score of 0 indicates that no structural features are shared in both structures. Conversely, a Tanimoto score of 1 indicates that all investigated structural features (determined by the fingerprint) are present in both structures. A Tanimoto score ≥0.8 indicates reasonable structural similarity, whereas scores ≥0.95 indicate very high structural similarity. 

The whole set of challenges was processed with the command line version of MetFusion. Results were stored in a structure data file (SDF), which is better known by the *.sdf file extension. This file keeps the molecular structure and associated information, like compound name, score, and additional properties, for each candidate. In addition to the integrated result list as an SD file, we also keep the individual intermediate result lists and create a spreadsheet file containing the result lists and the coloured similarity matrices which can be used to examine the results in more detail. 

## 3. Results and Discussion

In this section we discuss the results of our resubmissions and note where and why they differ from the original submissions. The challenges 2, 4, 5 and 6 from category 2 were not calibrated when initially offered to the participants, resulting in higher than stated ppm deviations. This was recognised after the contest closed, and the data of these challenges was recalibrated and made available to the participants online for the articles in the proceedings. Each participant was allowed to resubmit their findings. Additionally, our hypotheses for the neutral mass of challenges 11 and 12 were wrong in the first submission. The correct neutral mass for challenge 12 could be extracted from the available meta-data that all participants had access to. Challenge 11 did not provide [M+H]+ ions, instead the [M-H_2_O]+ fragment was the major ion suitable for back-tracking the neutral mass by an experienced mass spectrometrist. We used the correct neutral mass from the published CASMI solution for challenge 11. 

For both MetFrag and MetFusion we report the number of candidates and the absolute rank for each challenge, and the median rank broken down to the natural compound and environmental challenges. The median is used because the distribution of ranks is heavily tailed and a few challenges with very poor ranking severely skew the mean values. In addition to the absolute rank, we also report the relative ranking position (*RRP_CASMI_*), defined as 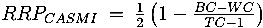 where *BC* and *WC* are the number of candidates ranked better and worse than the correct solution, and *TC* is the number of total candidates, respectively. See [16] for more details. 

### 3.1. MetFrag

In the initial submission, the correct solution was missing for Challenges 2, 4 and 6 because the measured mass was outside the 5 ppm margin. In addition, the simple precursor heuristics described in Section 2.1 missed the neutral mass of challenges 11 and 12. These cases were corrected with the updated information for the resubmissions. 

Table 1 shows the number of candidates obtained from the PubChem snapshot with a search for the neutral mass and the absolute rank of the correct solution. For Challenges 1 to 6 we also show the ranks with the MLS score included. 

**Table 1:** MetFrag results with neutral exact mass filter after resubmission. Shown are the number of candidates per challenge(#Cand.), the InChiKey filtered MetFrag rank and the relative ranking position (RRP). Additionally, for challenges 1–6 the InChiKey filtered MetFrag rank with the metabolite-likeness score (MLS) included is shown.

Natural Product Challenges						Environmental Challenges			
Chall.	#Cand.	Rank	RRP	MLS	RRP	Chall.	#Cand.	Rank	RRP
						10	447	260	0.441
1	994	5	0.996	4	0.997	11	465	23	0.976
2	248	3	0.992	3	0.992	12	1531	36	0.978
3	1094	12	0.990	9	0.993	13	1031	5	0.998
4	2234	547	0.757	454	0.797	14	125	27	0.810
5	2891	988	0.679	1238	0.573	15	1825	173	0.907
6	1860	1860	0.439	281	0.850	16	1948	1948	0.453
						17	475	15	0.970
Median	1477	280	0.874	145	0.921		753	32	0.939

The results achieved with the molecular formula database query are shown in Table A1. For every challenge MetFrag found the correct hit among the candidates with both types of queries, where the mass window result sets contain twice as many candidates. The absolute ranks obtained with the formula query decrease the median rank (Challenges 1–6: 280⇒270; Challenges 10–17: 32⇒22.5) compared to the ranks of the mass query, but on the other hand the median RRP is lower (Challenges 1–6: 0.874⇒0.607; Challenges 10–17: 0.939⇒0.917) with the use of the molecular formula filter, because compounds within the mass search window but with the wrong molecular formula often obtain a lower MetFrag score compared to the correct solution. The molecular formula filter eliminates these worse candidates (WC) from the outset, which reduces the RRP. 

Next, we describe the outcome if the metabolite-likeness score is considered together with the MetFrag score for the Challenges 1–6. The number of candidates remains unchanged, but natural compounds (including the correct solution) should obtain better scores and improve both the absolute rank and the RRP. 

Indeed, except for Challenge 5 all ranks are better or equal with the MLS contribution in the score as shown in Table 1. The median absolute rank decreases from 280⇒145 (RRP: 0.874⇒0.921) and even more for the molecular formula candidate search, where the median rank improves from 270⇒119 (RRP:0.607⇒0.797). 

Reticuline (the correct candidate of Challenge 5) has the lowest metabolite-likeness score of 0.296 among all challenge compounds and therewith the worst rank (1209) related solely to the MLS (see Table 2), which explains why the final result for Reticuline was even worse with MLS. 

**Table 2:** The metabolite-likeness score (MLS) of the compounds of Challenges 1 – 6 and their rankings among the retrieved candidates based on the MLS alone, while Table 1 uses he combined score.

Challenge	Trivial name	InChIKey (first block)	MLS	MLS rank
1	Kanamycin A	SBUJHOSQTJFQJX	0.508	47
2	1,2-Bis-O-sinapoyl-beta-D-glucoside	KQDOTXAUJBODDM	0.716	35
3	Glucolesquerellin	ZAKICGFSIJSCSF	0.474	3
4	Escholtzine	PGINMPJZCWDQNT	0.436	439
5	Reticuline	BHLYRWXGMIUIHG	0.296	1209
6	Rhoeadine	XRBIHOLQAKITPP	0.374	132

**Figure A1:**
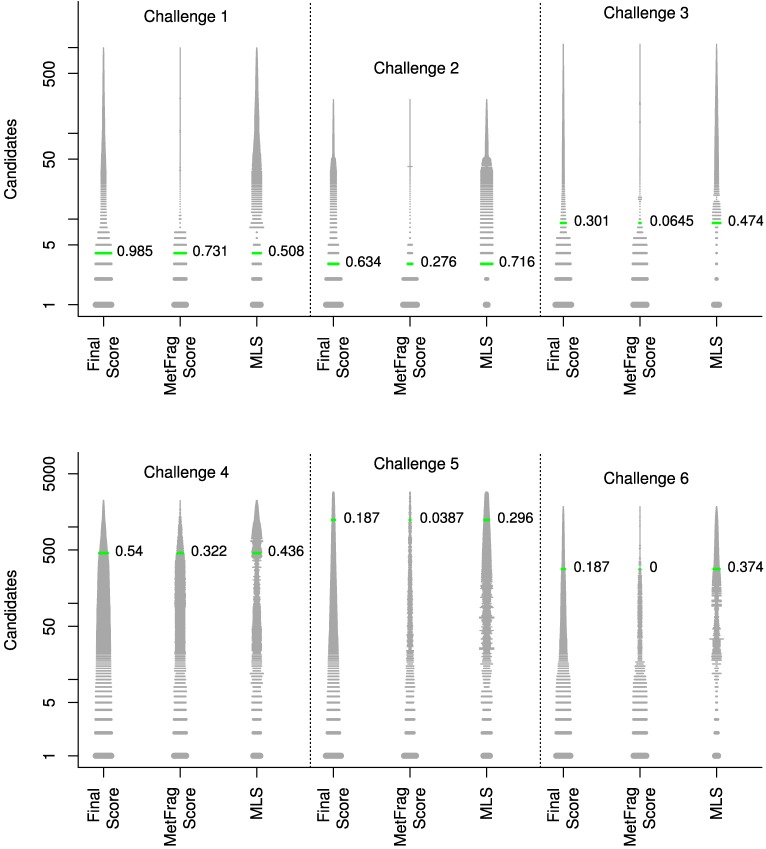
Scores plot of challenges 1–6. The MetFrag and metabolite-likeness score (MLS) as well as the final scores of the candidates are shown for the challenges, respectively. The green line marks the position of the correct candidate and the given score. The width of each line correlates with the represented value of the score, respectively.

Challenges 6 and 16 were very problematic for MetFrag, which could only assign to the given spectrum a single fragment of the correct molecule for the first case and no fragments of the correct molecule for the second case. Although the MLS improved the final rank for challenge 6, this is only based on the (second lowest among all challenges) MLS of 0.374. Figure A1 shows the rankings related the the calculated scores of all candidates of challenges 1 to 6. 

The results show that MetFrag is able to rank four molecules of the total 14 challenges among the top ten hits when applying mass filtering. The number can be increased to five by including knowledge of the molecular formula of the correct compound. 

The external participants Dunn *et al.* [17] and the internal participant Meringer *et al.* [19] both used MetFrag in conjunction with other methods for the identification. The combined MetFrag and manual interpretation method of Dunn *et al.* had better ranks than MetFrag alone, but missed a lot more challenges because the Kyoto Encyclopedia of Genes and Genomes (KEGG) [18] was used for candidate retrieval, which only contains a subset of the challenge compounds. 

### 3.2. MetFusion

The overall results for MetFusion are shown in Table 3. PubChem has grown considerably over the past two years and consequently the online query against PubChem yields more candidates: for the first six challenges, MetFrag retrieved 1477 candidates (median) from our PubChem snapshot (September 2010), whereas the corresponding online query against PubChem from January 2013 yields 3582 candidates (median)— more than twice as many, and more than three times for the environmental challenges. The same observation can be made for the remaining challenges 10–17. The rapid growth of PubChem over even short time periods becomes obvious; e.g., for Kanamycin A. In January 2013, 37 isomers with an identical first block of their InChIKey were retrieved, whereas only eight weeks later three additional isomers were found. 

**Table 3:** MetFusion results per challenge after resubmission. Shown are number of candidates per challenge (#Cand.), the InChIKey filtered MetFusion rank as well as the maximum Tanimoto similarity (Max. TS) between the candidates and the MassBank results and finally the relative ranking position (RRP).

Natural Product Challenges					Environmental Challenges				
Chall.	#Cand.	Rank	Max. TS	RRP	Chall.	#Cand.	Rank	Max. TS	RRP
					10	1085	981	0.40	0.096
1	2229	1	1.0	1.0	11	1444	170	0.28	0.883
2	625	4	0.93	0.995	12	3772	136	0.28	0.964
3	2945	14	0.99	0.995	13	3344	1	1.0	1.0
4	4219	74	0.84	0.983	14	507	3	1.0	0.996
5	4280	1426	0.42	0.667	15	3394	1	1.0	1.0
6	6175	25	0.79	0.996	16	4427	1351	0.33	0.695
					17	1848	88	0.35	0.953
Median	3582	20	0.89	0.995		2596	112	0.38	0.959

The results for challenges 1 to 6 and challenges 10 to 17 show that more similar spectra are present in MassBank for the biological compounds than for the environmental challenges. The median Tanimoto similarity between the challenges and the most similar compound in MassBank is 0.89 for the natural compounds, compared to 0.38 for the environmental challenges where the reference spectra did not contribute significantly to the integrated MetFusion score in five cases. This can be attributed to a much larger chemical diversity of natural products in MassBank. This is also evident by the low maximum spectral similarity. The lack of reference spectra for diverse non-biological compounds is the major reason for the mediocre performance of MetFusion in these cases. We expect a considerable improvement in this area as contributions to MassBank from the environmental community have recently increased. 

**Figure 1:**
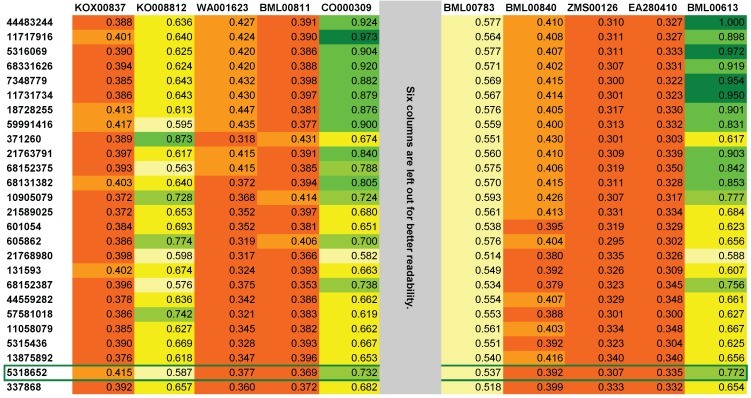
The top-left part of the reranked similarity matrix from MetFusion for Challenge 6. The correct compound rhoeadine is ranked 25th (CID 5318652) and is highlighted with a green border. The maximum Tanimoto similarity (TS) for rhoeadine has bicuculline with a similarity of 0.79, but a MassBank score of only 0.3 (data not shown). There are other alkaloids with better similarity that are thus ranked higher. Six columns were removed for better readability, altogether with a low maximum TS of 0.4.

**Figure 2:**
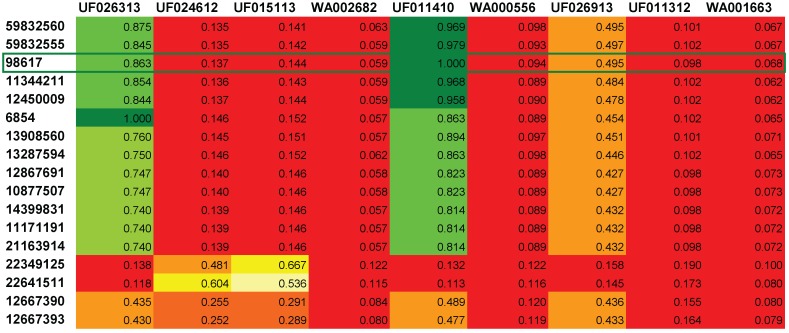
Excerpt of reranked similarity matrix from MetFusion for Challenge 14. The correct compound is ranked 3rd (CID 98617) and highlighted with a green border. The two better ranking candidates have slightly higher MetFrag scores that add to their corresponding MetFusion scores. Compound 6854 is carbazole, a structurally highly similar compound towards the correct 1H-Benz[g]indole. The presence of Tanimoto similarities with value of 1.0 indicate perfect structural matches according to corresponding reference spectra available in MassBank for both 1H-Benz[g]indole (UF011410) and carbazole (UF026313).

In addition to the ranked list of candidates, MetFusion also creates a ranked similarity matrix, where the columns correspond to the result list from MassBank (best hits on the left, ordered by the MassBank score) and the rows correspond to the MetFrag results. Each cell contains the Tanimoto similarity (TS) of the corresponding structures from MassBank and MetFrag. Examples are shown in Figure 1 and Figure 2. Tanimoto similarities are also visualised through a colour code ranging from red via yellow to green with increasing TS. 

Overall, MetFusion was able to rank the correct candidate in the top position for the three challenges 1, 13 and 15. Challenges 2 and 14 had the correct compound ranked at position 4 and 3, respectively. 

For Challenge 6, using MetFrag alone have a very poor result because 3812 candidates had an identical score of 0.0. MassBank does not contain spectra for the correct compound rhoeadine, and the most similar spectrum returned is palmatine (KOX00837), with a low 0.42 TS to the correct structure (as shown in Figure 1), while the structurally most similar entry (bicuculline, TS = 0.79) in MassBank has a poor spectral score of only 0.3. The main contribution from the MassBank results are three spectra from other alkaloids (allocryptopine, noscapine, and hydrastine) with a similarity between 0.59 and 0.77. 

For Challenge 14, shown in Figure 2, MassBank returned a spectrum of carbazole ranked first, an isomer of the correct 1H-Benz[g]indole, followed by three spectra of compounds with both a different molecular formula and lower TS than the MetFrag candidates. During the contest, spectra of the correct 1H-Benz[g]indole measured on the same instrument as the challenge data were submitted to MassBank by one of the MassBank consortium members. The UF011410 hit in MassBank was only ranked fifth, with an unexpectedly low MassBank score of only 0.70, most likely because we used a merged query spectrum and MassBank applies a 5% intensity cut-off. These two factors led to a greater difference between the merged spectrum and the deposited reference spectrum. The available Orbitrap spectra would benefit from a lower cut-off threshold of 2 rather than 5, but we relied on the default cut-off. With this low spectral similarity, the MassBank contribution was unable to lift the correct compound to the first rank, but only to rank 25. 

For challenges 1 to 6 MetFusion performed significantly better than MetFrag, and the median rank of the correct compound was 20, compared to 280 with MetFrag and 145 with MLS. This is even more remarkable because we used the online PubChem query, which returned 3145 candidates (median), whereas the PubChem snapshot only provided 1063 candidates (median) over all challenges. 

MetFusion results for challenges 10 to 12 were significantly worse when compared to MetFrag alone. This can be attributed to the low Tanimoto similarity of the correct candidate to any of the spectral hits. For each of these challenges, the MassBank scores are between 0.31 and 0.68 for the top hit, indicating a lack of reference spectra for these compound classes. The missing spectral coverage is expressed in both mediocre spectral scores and almost no Tanimoto similarity, visualised by the red-orange coloured matrix cells with maximum Tanimoto similarity of 0.4. This indicates the case where the spectral library cannot confirm any of the *in silico* candidates, thus leaving the user with no additional information. 

## 4. Conclusions

The IPB entered the CASMI contest unofficially, because as part of the organising team and challenge data providers we could not be considered independent. However, we entered CASMI as internal participants with MetFrag and MetFusion and did not tune the parameters to obtain optimal results for the initial submission. 

The use of small, domain-specific compound databases like KEGG, focussing on natural compounds increases the risk that the correct compound is missed. While such a compound may be more likely to be found in PubChem or ChemSpider, the number of false positives will increase due to the large number of synthetic compounds. We used the metabolite-likeness score [11] as an additional term in the scoring function of MetFrag. The metabolite-likeness score penalizes synthetic compounds and improved the rankings for the natural product challenges 1–6 in all but one case. Moreover, we see potential for further improvement of these preliminary results by optimisation of the weight factor *ω* and the evaluation on a larger dataset than available in the CASMI contest. 

MetFusion was used without additional scoring terms, such as the metabolite-likeness score. The similarity matrices provide a deeper insight into the integrated MetFusion score to (manually) assess the reliability of the MassBank spectral summary. 

Both approaches were applied fully automatically to the challenge data, but the selection of the neutral mass for the candidate failed in two cases, and the scoring did not always rank the correct solution in the top positions. Although expert knowledge is still required for a reliable interpretation, our approaches can reduce the manual effort for small compound identification. 

We are looking forward to participating in the next CASMI contest as external participants. 

## Appendix

### A. Additional MetFrag Results

**Table A1:** MetFrag results with molecular formula filter after resubmission. Shown are the number of candidates per challenge, the InChIKey filtered MetFrag rank and the relative ranking position (RRP). Additionally, for challenges 1-6 the InChIKey filtered MetFrag rank with the metabolite-likeness score (MLS) included is shown.

Natural Product Challenges						Environmental Challenges			
Chall.	#Cand.	Rank	RRP	MLS	RRP	Chall.	#Cand.	Rank	RRP
						10	257	170	0.377
1	9	5	0.500	4	0.625	11	104	9	0.961
2	43	1	1.000	1	1.000	12	950	26	0.975
3	2	2	0.500	1	1.000	13	22	4	0.929
4	2005	534	0.735	444	0.779	14	111	19	0.859
5	2429	754	0.714	920	0.623	15	1789	172	0.905
6	1250	1250	0.416	234	0.814	16	1397	1397	0.438
						17	415	15	0.966
Median	646	270	0.607	119	0.797		336	22.5	0.917
